# Phytochemical Composition of Different Red Clover Genotypes Based on Plant Part and Genetic Traits

**DOI:** 10.3390/foods13010103

**Published:** 2023-12-28

**Authors:** Mira Mikulić, Milica Atanacković Krstonošić, Nebojša Kladar, Sanja Vasiljević, Snežana Katanski, Zlatica Mamlić, Dušan Rakić, Jelena Cvejić

**Affiliations:** 1 Department of Pharmacy, Faculty of Medicine, University of Novi Sad, Hajduk Veljkova 3, 21000 Novi Sad, Serbia; mira.bursac@mf.uns.ac.rs (M.M.); nebojsa.kladar@mf.uns.ac.rs (N.K.); jelena.cvejic@mf.uns.ac.rs (J.C.); 2 Institute of Field and Vegetable Crops, National Institute of the Republic of Serbia, Maksima Gorkog 30, 21000 Novi Sad, Serbia; sanja.vasiljevic@ifvcns.ns.ac.rs (S.V.); snezana.katanski@ifvcns.ns.ac.rs (S.K.); zlatica.miladinov@ifvcns.ns.ac.rs (Z.M.); 3 Department of Basic Engineering Disciplines, Faculty of Technology, University of Novi Sad, Bulevar cara Lazara 1, 21000 Novi Sad, Serbia; drakic@tf.uns.ac.rs

**Keywords:** *Trifolium pratense*, isoflavones, ploidy, HPLC, total phenolic content, antioxidant activity

## Abstract

Red clover (*Trifolium pratense* L.) is an important legume that is also known as a rich source of isoflavones, which are compounds with mild estrogenic activity. Therefore, this plant is often used as a raw material in the production of dietary supplements recommended in menopause. Many factors can influence isoflavone content, but those genetically related are considered to be the most important. Therefore, the aim of this study was to evaluate the phytochemical profile of different plant parts of 30 red clover genotypes grouped according to ploidy and country of seed origin by analyzing the content of dominant isoflavones, total phenolic content (TPC) and antioxidant activity. It was found that there are significant differences in the examined traits among plant parts. Red clover leaves had the highest total isoflavone content, with biochanin A as the dominant compound, while flower extracts had the highest TPC and antioxidant activity. Diploid and tetraploid genotypes were significantly different concerning the content of daidzein, genistein, formononetin and TPC with higher quantities in tetraploid samples. On the other hand, seed origin was not a useful separating factor for the analyzed samples. The results of this research indicate that ploidy, as a previously poorly studied factor, could influence isoflavone content in red clover.

## 1. Introduction

Red clover (*Trifolium pratense* L.) is one of the most important legumes used for forage, and it is cultivated in regions with temperate climate mainly for its high-protein feed. Due to its biological nitrogen fixation ability, red clover has been recognized as an important species from the aspect of sustainable farming [1,2]. Although it is commonly used for animal feed, recently, it became very popular in the food, pharmaceutical and cosmetics industries as a valuable source of bioactive compounds [3]. Fresh or cooked red clover leaves and flowers can be consumed as a part of healthy diet in addition to salads or in a traditional way as tea or syrup. Red clover sprouts are also considered as “super” food due to their high mineral and vitamin content. Namely, red clover is known for its high isoflavone content. Isoflavones found in this plant exert mild estrogenic effect in mammals due to their structural similarity to 17β-estradiol, which includes them in the group of phytoestrogens. At first, negative effects in animals were observed, since feed rich in isoflavones disrupted reproductive cycles in sheep [4,5,6]. Later, when specific compounds responsible for this biological activity were identified, it was clear that red clover isoflavones could be used as a natural estrogen substitute primarily by menopausal women. Therefore, red clover found its place in the pharmaceutical industry as a raw material used for dietary supplement production.

**Figure 1:**
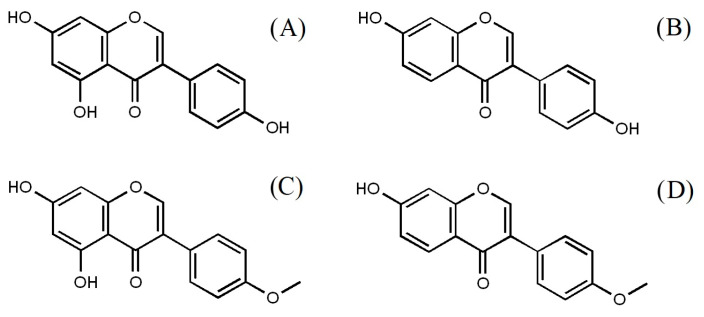
Chemical structures of red clover isoflavones: (**A**) genistein, (**B**) daizein, (**C**) biochanin A, (**D**) formononetin.

The predominant isoflavones in red clover are usually biochanin A, formononetin, daidzein and genistein (Figure 1) together with some other aglycone compounds such as glycitein, irilone, orobol, pratensein, pseudobaptigenin, and prunetin, which are present in lower concentrations. Biochanin A and formononetin are actually 4-methyl ether derivatives of genistein and daidzein, respectively. Glucoside isoflavone forms (e.g., sissotrin, ononin) are found in much smaller amounts compared to aglycone forms [7,8,9]. Comparing the binding affinity of different isoflavones to estrogen receptors (ER), which is one of the main mechanisms of their biological activity, it was found that it increases in the following order: genistein, daidzein, biochanin A, and formononetin. Additionally, it was proved that by combining formononetin with β-estradiol, a strong synergistic binding effect to ERβ can be achieved, implying that supplementation with formononetin can be beneficial before as well as after menopause [10,11].

Formononetin has been recognized as a neuroprotective compound that is valuable for the treatment of Parkinson’s disease [12]. Additionally, there are numerous in vivo and in vitro studies proving its anti-cancer potential by various mechanisms [13]. Biochanin A also shows a plenitude of biological activities such as hypoglycemic, anti-microbial, anti-cancer, anti-oxidant, as well as osteogenic and anti-inflammatory properties [14,15]. Other polyphenolic compounds, such as flavonoids, polyphenolic amides and phenolic acids, which are present in red clover, can also contribute to its antioxidant potential [7,16,17].

Nowadays, various red clover dietary supplements recommended mainly for the alleviation of menopausal symptoms are present on the market. There is clinical evidence on the efficacy of the standardized extract of red clover isoflavones (Promensil^®^), containing biochanin A, formononetin, genistein and daidzein, at a 80 mg/day dose, in the treatment of hot flashes [18]. Additionally, red clover powder extracts, which can be added to food or used as a beverage, are available to consumers and used for weight gain, lowering cholesterol levels and aiding in the prevention of osteoporosis.

Although red clover is one of the richest sources of isoflavones in nature, it is not often present in its raw form as a part of human nutrition. Nevertheless, the consumption of red clover sprouts is becoming more popular, for example as an addition to salads or as a garnish. Both leaves and flowers are also edible and have a gentle and sweet taste when eaten raw. There are several other food sources of similar isoflavones such as different kinds of beans, chickpeas, alfalfa seeds, eggs, apricots, grapefruit, cabbage, peanuts, etc. [15,19]. Soybeans and soy products are actually major sources of isoflavones in the human diet, with daidzein and genistein being the most dominant compounds, but with only small amounts of formononetin. In soy, isoflavones are mainly present in glucoside forms, while in red clover, aglycones are dominant [7]. It is known that glucosides usually have to be hydrolyzed in the gut before their absorption; thus, it is considered that aglycones have higher bioavailability in humans [11,20]. Additionally, it seems that isoflavones in methylated forms (formononetin and biochanin A), dominant in red clover, are better absorbed than the corresponding unmethylated isoflavones (daidzein and genistein) [21,22]. Also, an increasing number of soy-related allergies as well as the skepticism of health-conscious consumers toward genetically modified soy led to a rise in interest in alternative sources of isoflavones [8].

Numerous studies indicate that the genotype is probably the most important factor influencing the production of isoflavones and other secondary metabolites in red clover [5,7,8,23,24]. Also, environmental conditions, climate, as well as plant phenophase can determine the content and composition of these compounds [7,25]. Selection for red clover with lower phytoestrogen levels has been effective and directed toward the breeding of genotypes suitable for animal nutrition [26]. On the other hand, the identification of plant materials with high concentrations of phytoestrogens or desirable ratios of specific isoflavones, combined with good agronomic performance, could progress the genetic improvement of red clover for various purposes [4,27]. In view of the great demand for high-quality raw materials in the production of novel foods, supplements or cosmetic products based on red clover isoflavones, it is desirable to investigate the effect of less studied factors on the content and composition of phytoestrogens in the plant. Therefore, the objective of this study was to analyze the isoflavone content and profile, total phenolic content and antioxidant activity in different plant parts of 30 red clover varieties in order to elucidate the influence of plant ploidy and seed origin on these important traits.

## 2. Materials and Methods

### 2.1. Reagents and Materials

Solvents used for extraction and HPLC analysis included the following: ethanol 96% (Zorka Pharm, Šabac, Serbia), hydrochloric acid 35% (POCH, Gliwice, Poland), methanol—HPLC grade, min 99.8% (J.T. Baker, Phillipsburg, NJ, USA), distilled water (TKA water purification system type 05.30C 7-DEN, Department of Pharmacy, Faculty of Medicine Novi Sad), sulfuric acid p.a. (RTB, Bor, Serbia) and acetonitrile—HPLC grade (J.T. Baker, Phillipsburg, NJ, USA). During total phenolic content determination, anhydrous sodium carbonate, min 99.5% (Sinex laboratory, Belgrade, Serbia), Folin–Ciocalteu phenolic reagent (Fluka Biochemika, Buchs, Switzerland) and gallic acid, 99% (Alfa Aesar Lancaster Synthesis, Ward Mill, MA, USA) were used. Antioxidant potential was tested with 2,2-diphenyl-1-picrylhydrazyl (DPPH) radical (Sigma Aldrich, Steinheim, Germany). Standard isoflavone substances were daidzein, genistein (ChromaDex, Los Angeles, CA, USA), formononetin and biochanin A (Sigma-Aldrich).

### 2.2. Red Clover Samples

Red clover samples were obtained from the Institute of Field and Vegetable Crops, Novi Sad, Serbia, originating from domestic institutes (Serbian commercial varieties) as well as from two donors (two plant gene banks: Nordic Gene Bank, Sweden, and IPK-Gatersleben, Germany). The plants were taken from the second cut (in the first half of July) in the stage of full flowering [23], during the first year of exploitation at the experimental field of the Institute of Field and Vegetable Crops in Rimski Šančevi, Serbia. After sampling, the green parts of the plant were physically separated into three groups—leaf, stem and flower. The samples were dried at 60 °C for about 48 h and then separately packed in paper bags and stored in a dry and dark place until analysis.

The experiment included 30 red clover genotypes, of which 20 are diploid (2n) and 10 are tetraploid (4n) with seeds originating from six different countries (Sweden, Germany, Slovakia, Serbia, Czech Republic and France) (Table 1). Since the leaf, flower and stem of each plant were analyzed separately, a total of 90 samples were analyzed.

### 2.3. Extraction

Previously homogenized and ground plant material (1 g) was mixed with 2 mL of water and incubated in a water bath for 30 min at 37 °C. Then, 16 mL of ethanol and 2 mL of 3M HCl were added, and the samples were heated to boiling with stirring for 10 min [23]. This procedure enables the hydrolysis of glucoside isoflavone forms to corresponding aglycones. After cooling, the extracts were filtered through filter paper. The obtained extracts were diluted with 96% ethanol when needed and used for the total phenolic content and the antioxidant capacity determination. The solid phase extraction (SPE) was used for the further purification of extracts before HPLC analysis. The SPE method proposed by Klejdus et al. (1999) was applied with slight modifications, using the SPE manifold 12G (J.T. Baker, Phillipsburg, NJ, USA). Oasis HLB cartridges 3cc (Waters, Milford, MA, USA) were conditioned with 3 mL of methanol and equilibrated with 3 mL of distilled water. Then, 0.5–1 mL of extract with 2 mL of water was added. Washing was completed with 3 mL of 5% methanol, and elution was completed with 3 mL of 80% methanol, 2 mL of 90% methanol and 5 mL of 100% of methanol [28]. Recoveries for all the compounds were above 90%. Prior to HPLC analysis, the obtained extracts were filtered through Agilent RC 0.45 µm filters.

### 2.4. HPLC Analysis of Isoflavones

Isoflavone separation was achieved on an Agilent (Palo Alto, CA, USA) model 1100 series HPLC equipped with a binary pump, degasser, auto sampler and diode array detector (DAD) with Zorbax SB C18 column (250 × 4.6 mm, 5 µm). The mobile phase consisted of solvent A (water adjusted to pH = 2.7 with sulfuric acid) and solvent B (acetonitrile). The following gradient program was applied: 0–35 min from 20 to 35% B, 35–45 min from 37 to 100% B, 45–50 min 100% B, 50–51 min from 100 to 20% B, 51–61 min 20% B, with a post time of 15 min 20% B. The flow rate was 1 mL/min, and the detection wavelength was 254 nm. The injection volume was 10 µL, and the analysis was performed at a constant temperature of 25 °C [29]. Isoflavones were identified based on the retention time and UV spectra of the corresponding standard substances—daidzein, genistein, formononetin, and biochanin A (Figure S1). The standards were dissolved in 80% methanol. For quantification, five-point calibration curves were constructed for each compound with a correlation coefficient of r^2^ ≥ 0.999. The isoflavone content was expressed in mg per g of dry weight (DW).

### 2.5. Total Phenolic Content (TPC)

The content of total phenolics was determined spectrophotometrically on an Agilent 8453 (Waldbronn, Germany) spectrophotometer by the Folin–Ciocalteu method using gallic acid as a reference standard [30]. The sample extract (0.1 mL) and Folin–Ciocalteu reagent (0.5 mL) were mixed well. After 6 min, 0.4 mL of saturated Na_2_CO_3_ solution (177 g Na_2_CO_3_/L) was added. The absorbance was measured at 740 nm after 120 min. The results were expressed as gallic acid equivalents per g of plant dry weight (mg GAE/g of DW), using a gallic acid standard curve.

### 2.6. Antioxidant Capacity

The free radical scavenging capacity (RSC) was evaluated spectrophotometrically after the reaction with 2,2-diphenyl-1-picrylhydrazyl (DPPH) radical [31]. For this purpose, 1 mL of ethanol DPPH solution (0.097 mM) was mixed with 20–400 μL of sample extract and diluted with 80% ethanol to 4 mL. The reduction in the DPPH radical was measured after 60 min at 515 nm. For every concentration of extract, the radical scavenging capacity (RSC) percentage was calculated using Equation (1):

(1) $$RSC=100-100\cdot \frac{A_{extract}}{A_{blank}}$$

where A_extract_ represents the absorbance of the analyzed sample extract and A_blank_ represents the absorbance of the blank sample. The calibration curve RSC versus extract concentrations were plotted, and the concentration of extract necessary to achieve an RSC value of 50% (IC_50_—inhibitory concentration) was expressed in mg of dry matter per mL.

### 2.7. Statistical Analysis

The obtained results were summarized by application of MS Office Excel (v2019) and further processed by application of Tibco Statistica (v13.5). The results were analyzed by descriptive statistical methods as well as by univariate and multivariate statistical methods. The correlation between the studied variables was estimated by Pearson correlation coefficient, while the differences between the analyzed groups were evaluated by application of the *t*-test and one-way ANOVA followed by post hoc Tukey’s HSD test. The level of significance was kept at *p* = 0.05. For the purpose of better describing the patterns of obtained dataset variability, Principal Component Analysis (PCA), as a dimension reduction technique, was also applied.

## 3. Results

### 3.1. Isoflavone Content

In the majority of the analyzed red clover samples, four isoflavones were quantified. Exceptions were several genotypes from France (m58, m59, m62 and m65), where daidzein was not determined in any plant part (Table 2). Generally, biochanin A was the dominant compound in most of the samples, which is usually followed by formononetin. Observed individually, the sample with the highest average total isoflavone content (5.71 mg/g) was Slovakian 4n genotype m82 due to the high biochanin A (5.49 mg/g) and formononetin (4.51 mg/g) content observed in leaves (Table 2). The sample with the lowest average total isoflavone content (0.67 mg/g) was m59 (2n genotype from France). Genotype m40 (4n from Germany) stood out because of the high genistein content in leaves (4.52 mg/g) and biochanin A content (1.52 mg/g) in flowers. Likewise, stems of sample m84 (2n, Czech R.) had the highest formononetin (1.54 mg/g) and biochanin A (0.56 mg/g) content (Table 2).

**Figure 2:**
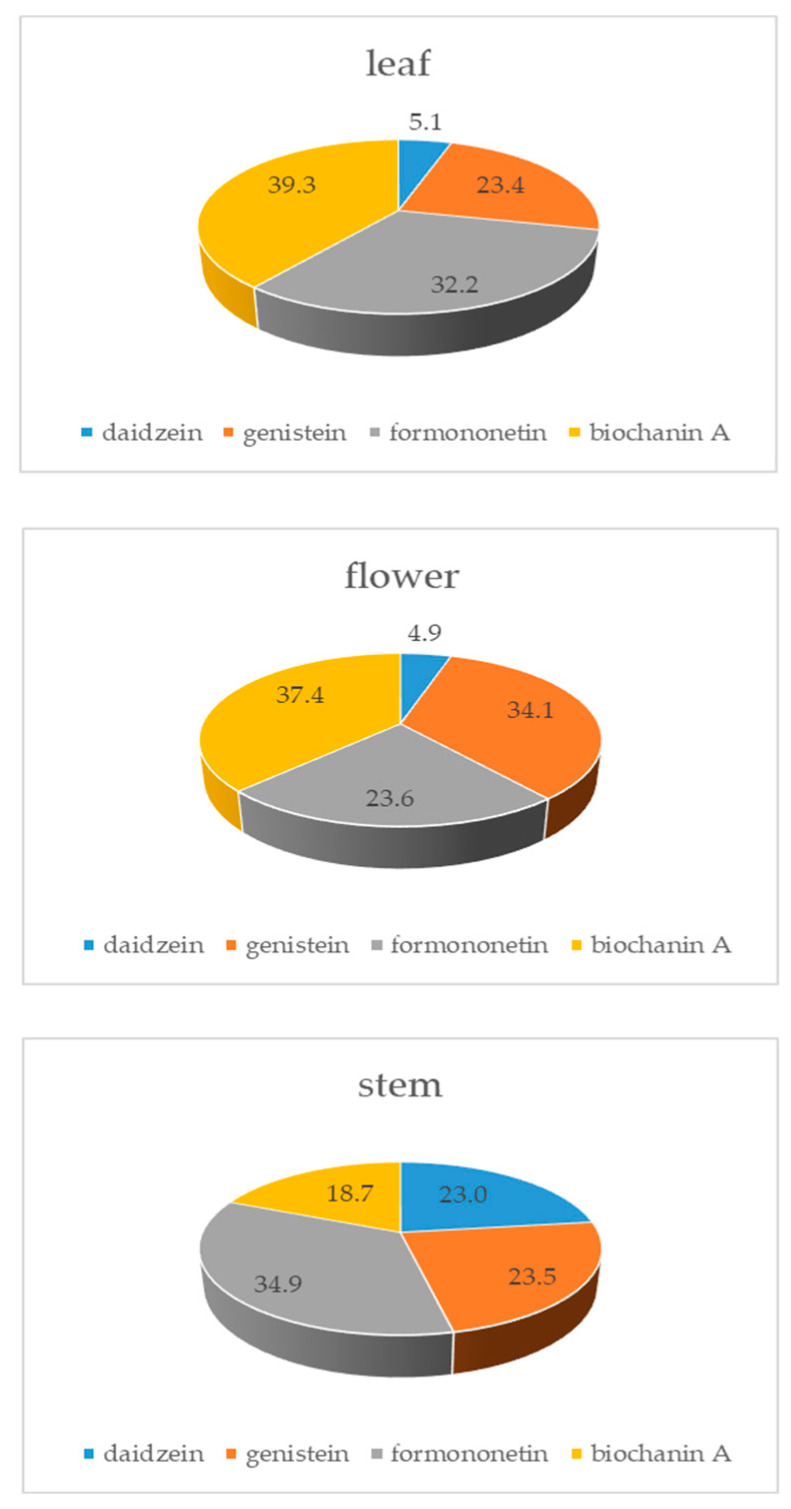
Distribution of individual isoflavones (%) in different red clover plant parts.

**Figure 3:**
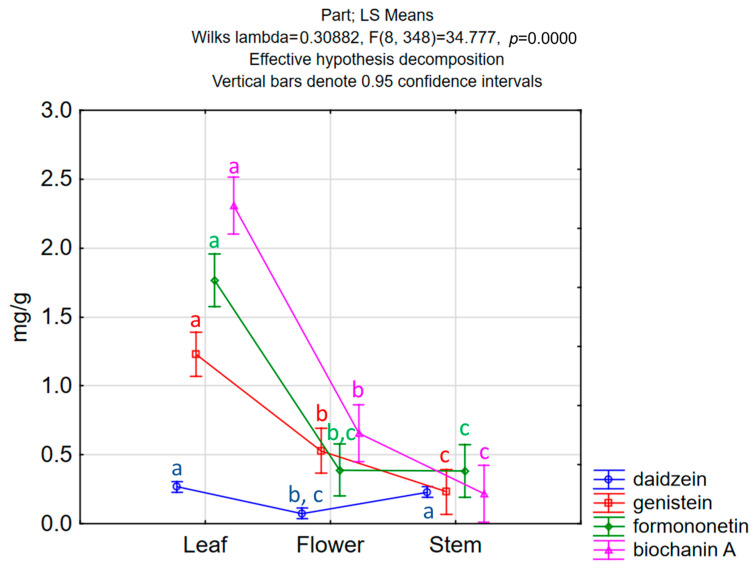
ANOVA results—differences in individual isoflavone content among plant parts; different small letters indicate statistically significant differences.

Leaves were on average the richest source of isoflavones (5.96 ± 3.04 mg/g), which were followed by flowers (1.78 ± 0.77 mg/g) and stems (1.10 ± 050 mg/g). In leaves, the total isoflavone content varied greatly among the red clover samples from 0.87 to 13.05 mg/g (Table 2). If the average distribution of individual isoflavones in the whole plant is observed, it can be noticed that biochanin A makes up over 36% of the total isoflavones, while the average percentage of formononetin is about 30%. In different plant parts, individual isoflavone distribution can vary (Figure 2). Leaves, having the highest total isoflavone content, dictate the average distribution in the whole plant, since biochanin A and formononetin make up over 70% of total isoflavones. In flowers, a high percentage of genistein is present (34.1%), with biochanin A having the highest content (37.4%). On the other hand, in stems, formononetin is the dominant isoflavone (34.9%), and there is a quite high share of daidzein (23%). Biochanin A is, surprisingly, the isoflavone with the lowest content in stems (18.7%) (Figure 2 and Figure 3). The application of ANOVA has demonstrated statistically significant differences in the quantified amounts of daidzein (F(177,2) = 26.71, *p* = 0.001), genistein (F177,2) = 39.37, *p* = 0.001), formononetin (F(177,2) = 68.27, *p* = 0.001) and biochanin A (F(177,2) = 11.54, *p* = 0.001) related to the analyzed plant parts, whereas the application of a post hoc Tukey’s HSD test indicated specific plant parts contributing to the recorded differences (Figure 3).

**Figure 4:**
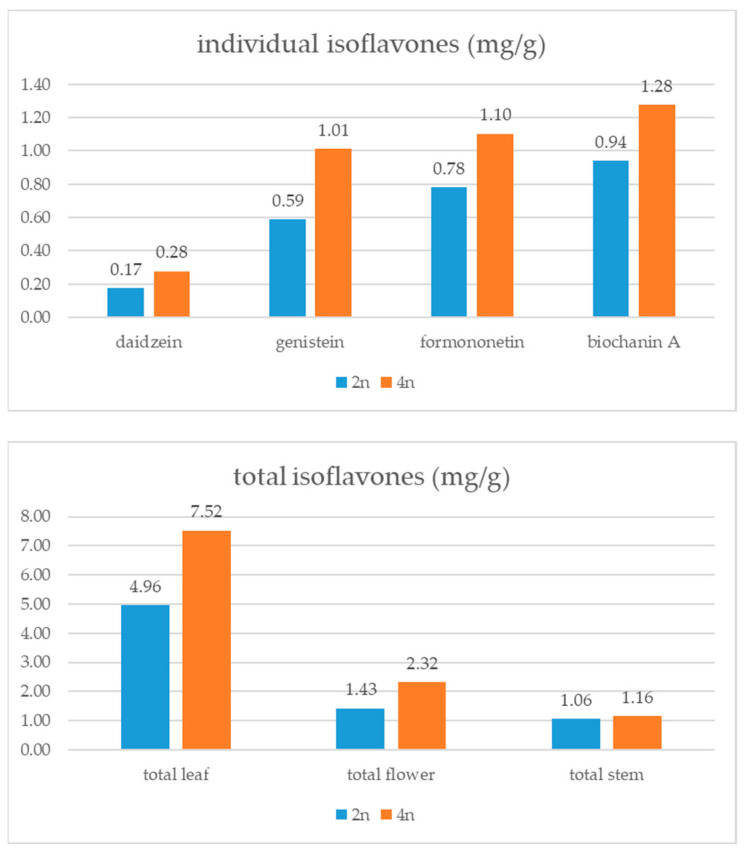
Isoflavone content (mg/g of DW) in red clover genotypes of different ploidy.

Concerning the ploidy of the analyzed samples, it can be noticed that 4n plants had, on average, higher amounts of all detected isoflavones. A similar trend was observed for the total isoflavone content in different plant parts. More specifically, the total isoflavone content in leaves and flowers of 4n samples was over 50% higher compared to its content in 2n samples (Figure 4). When a *t*-test was applied, it was established that 2n and 4n cultivars are significantly different concerning the daidzein (t(178) = −5.02, *p* = 0.001), genistein (t(178) = −4.62, *p* = 0.001) and formononetin (t(178) = −2.49, *p* = 0.010) content. On the other hand, the profile of individual isoflavones was on average very similar in the 2n and 4n samples (Figure 4).

**Figure 5:**
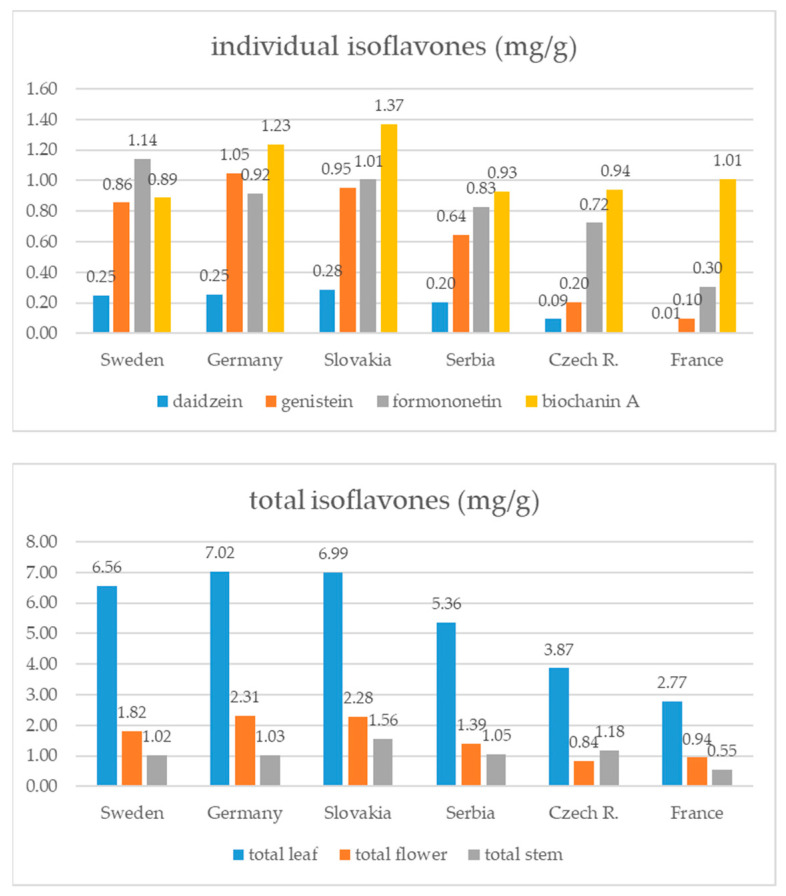
Isoflavone content (mg/g of DW) in red clover genotypes with different seed origin.

According to the country of seed origin, the red clovers from Slovakia and Germany had the highest average total isoflavone content (3.61 ± 2.94 and 3.45 ± 2.18 mg/g, respectively), while the lowest isoflavone content was noticeable in the samples from France (1.42 ± 1.18 mg/g). Generally, the individual isoflavone share follows the same order—biochanin is the dominant compound, which is followed by formononetin, genistein and daidzein. The contribution of individual isoflavones differs in samples originating from Sweden and Germany, since in the Swedish samples, formononetin is the dominant isoflavone, and in the German genotypes, the content of genistein is higher than the formononetin content (Figure 5). Concerning the plant part, generally, leaves were the richest sources of isoflavones and stems had the lowest content regardless of the country of seed origin. Only samples from the Czech Republic had the lowest isoflavone content in flowers compared to other plant parts (Figure 5).

### 3.2. Total Phenolic Content (TPC) and Antioxidant Capacity

Generally, it can be noticed that stems have lower TPC values as well as antioxidant potential compared to other plant parts. ANOVA showed that TPC and IC_50_ values are different in all the analyzed plant parts (F(2.177) = 125.07, *p* = 0.001; (F(2.177) = 574.23, *p* = 0.001), respectively), whereas a post hoc test indicated that differences exist between the TPC content and antioxidant activity of stems compared to leaves and flowers. The lowest IC_50_ values were observed in flower extracts, meaning that they have the highest average antioxidant potential (0.099 ± 0.035 mg/mL). At the same time, flower extracts contained the highest amount of total phenolics (28.52 ± 9.74 mg GAE/g on average). Sample m40 (4n, Germany) demonstrated the highest TPC value in leaf (38.38 mg GAE/g) and flower (47.05 mg GAE/g) extract as well as the lowest IC_50_ value in flower extract (0.059 mg/mL) (Table 3). Also, it can be observed that the sample m21 (4n, Sweden) had the highest TPC (16.84 mg GAE/g) and antioxidant potential (0.228 mg/mL) among stem extracts. Samples originating from France had the lowest TPC in all plant parts (Table 3).

**Figure 6:**
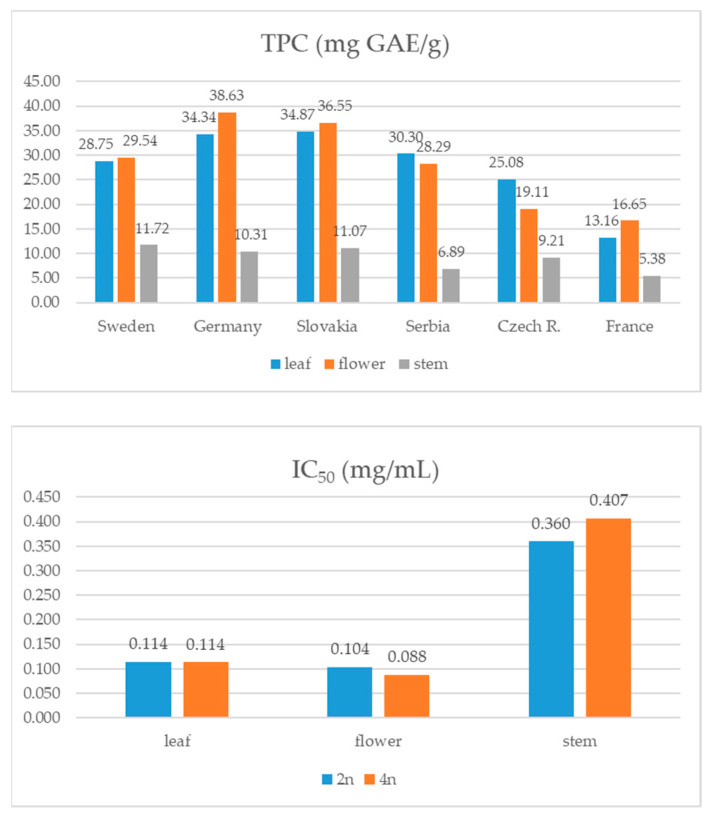
Total phenolic content (mg GAE/g of DW) and antioxidant activity (IC_50_ mg/mL) of red clover genotypes with different ploidy.

**Figure 7:**
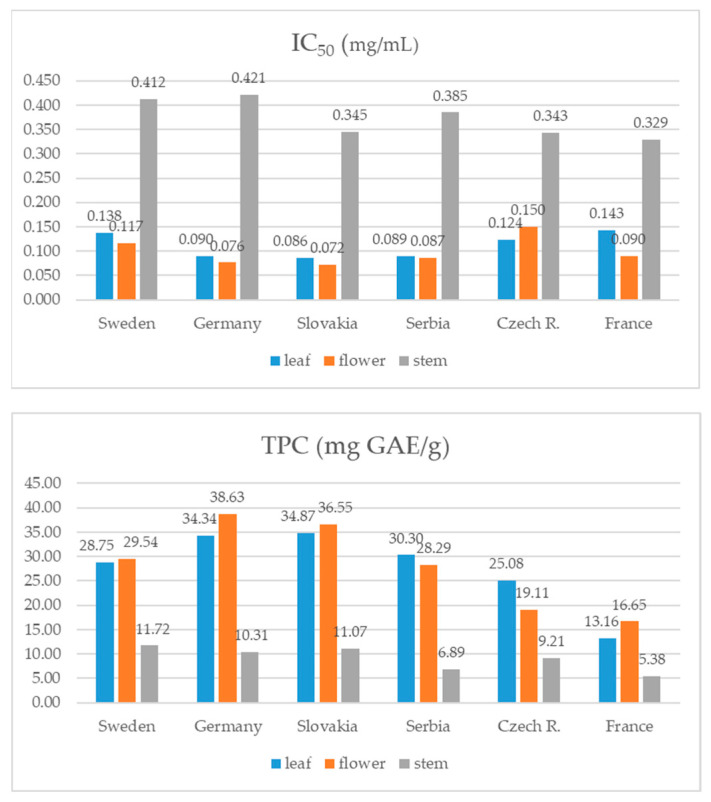
Total phenolic content (mg GAE/g of DW) and antioxidant activity (IC_50_ mg/mL) of red clover genotypes with different seed origin.

Similar to the total isoflavone content, different plant parts of the 4n samples had on average higher TPC values compared to the 2n samples. Statistical analysis confirmed that the 2n and 4n samples have significantly different TPC values (t(178) = −4.38, *p* = 0.001). This difference was not observed for IC_50_ values (Figure 6). Results for TPC in the samples originating from different countries showed that the German and Slovakian plants had the highest phenolic content (around 28 mg GAE/g), while the lowest was determined for the French samples. The samples from Slovakia also had the highest average antioxidant potential (0.168 ± 0.15 mg/mL), while the samples from other countries did not follow the same pattern observed for TPC (Figure 7). On the other hand, stems had obviously the lowest antioxidant potential compared to other plant parts regardless of the genotype origin. The samples from Serbia and the Czech Republic have the highest TPC in leaves, while this value was on average the highest in flowers of plants from other countries. When statistical analysis was performed, no significant differences concerning IC_50_ and TPC values were observed among the samples with different seed origin.

**Figure 8:**
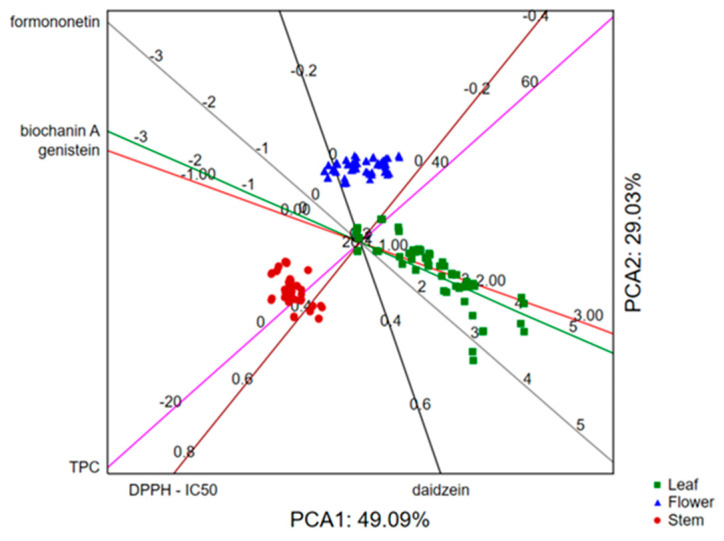
Principal Component Analysis of examined traits in different red clover plant parts.

The application of PCA on the dataset describing the phytochemical profile of the analyzed samples in terms of total phenolic content and quantities of formononetin, biochanin A, genistein and daidzein, as well as antioxidant potential (expressed as IC_50_ value) by taking into account the analyzed plant part, shows that the first two principal components describe around 80% of the samples’ variability. The size of the variability mostly correlates to the recorded antioxidant potential of samples, the content of formononetin and biochanin A, as well as the total phenolics content. The position of the analyzed samples and variables in the space defined by the first two principal components (Figure 8) indicates separative grouping of the stem extracts as a result of the lower antioxidant potential and total phenolics content. On the other hand, the flower extracts were the most potent scavengers of DPPH radicals, while the leaf extracts contained the highest quantities of biochanin A and formononetin.

**Figure 9:**
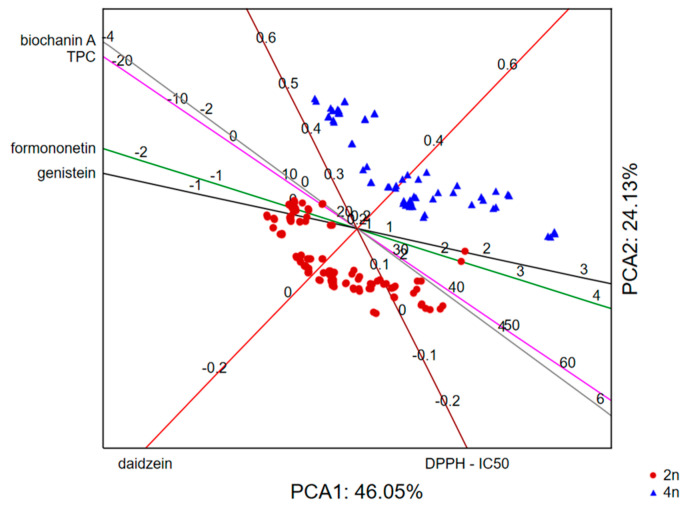
Principal Component Analysis of examined traits in red clover genotypes of different ploidy.

Furthermore, if we process the same dataset by taking into the account the average values of the examined variables in three plant parts and the ploidy of the analyzed samples (2n/4n), the results show that the first two principal components describe around 70% of the samples’ variability. The recorded variability mostly correlates with biochanin A and genistein quantities as well as with antioxidant potential. However, the position of the analyzed samples in the space defined by the first two principal components (Figure 9) indicates daidzein as an important marker responsible for the separative grouping of 2n and 4n samples with quantities higher in 4n samples.

**Figure 10:**
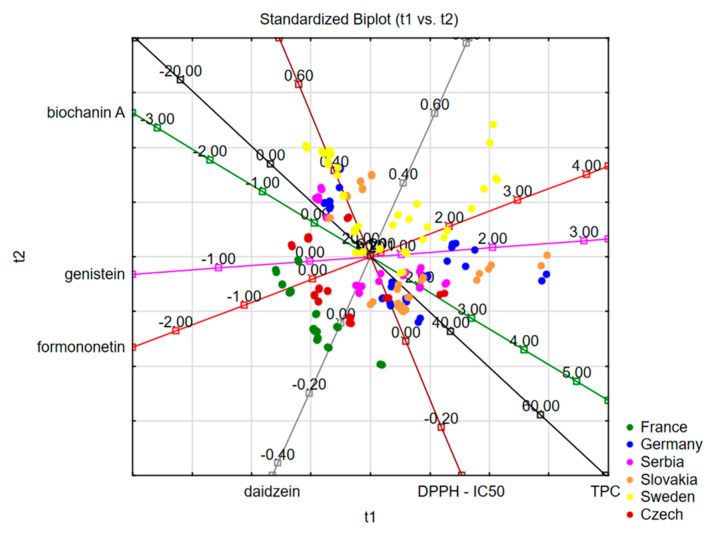
Principal Component Analysis of examined traits in red clover genotypes with different seed origin.

When the data for isoflavone content, antioxidant activity and TPC were analyzed according to different countries of red clover seed origin, there was no separative grouping of the studied samples. However, it can be observed that the samples from Sweden and the samples originating from France belong to two separate groups when all tested parameters are taken into account (Figure 10).

Correlation analysis showed that there is a strong negative correlation between TPC and IC_50_ values (r = −0.80, *p* < 0.05). Furthermore, the TPC and antioxidant activity correlate with genistein, formononetin and biochanin A content (*p* < 0.05).

## 4. Discussion

The isoflavone content in red clover was investigated by several authors in the last two decades. The content of these compounds varies greatly depending on different factors. However, the majority of studies found that formononetin and biochanin A are the dominant isoflavones in red clover [32,33,34,35,36,37,38,39]. In this study, concentrations of formononetin were in ranges of 0.22–4.68 mg/g, 0–0.95 mg/g and 0.10–1.54 mg/g in leaves, flowers and stems, respectively, while these values for biochanin A were 0.59–5.94 mg/g, 0.17–1.52 mg/g and 0.08–0.56 mg/g (Table 2). These ranges correspond with several published results [5,23,32,33,36,37]. Still, higher values are also observed and can go up to 28.5 mg/g for formononetin [38] and 17.9 mg/g for biochanin A [7]. Also, it was established that leaves contain the highest concentration of isoflavones, which corroborates with the results of this study (Table 2). Concerning other plant parts, in a few studies, it is reported that flowers contain the lowest concentration of isoflavones [7,23,33,37]. On the other hand, a study published by Butkutė et al. (2014) showed that stems had the highest total isoflavone concentrations (over 4.5 mg/g) among aerial plant parts. Furthermore, it was shown that plant roots can also be a rich isoflavone source [24]. Other factors that seem to influence the isoflavone content and composition in red clover are plant maturity or growth stage [23,35,39] and time of harvest [5]. Tsao et al. (2006) established that in all parts of the red clover plant, the isoflavone content was higher in the later flowering stage compared to the early bud stage [7]. It is also known that isoflavones are phytoalexins, which are protective compounds synthesized in plants as a reaction to biotic and abiotic stress; therefore, poor weather conditions can increase their concentration [5].

Red clover is a diploid species by its nature, with seven chromosomes (2n = 2x = 14), while commercially, it is grown as a diploid or tetraploid cultivar. Breeders obtain cultivars with a tetraploid set of chromosomes (2n = 4x = 28) using several methods (colchicine doubling, N_2_O and sexual polyploidization through unreduced gametes), which is mainly because of some improved traits of 4n cultivars such as increased disease resistance, persistence and forage yield [26,40,41]. From the agricultural aspect, some of the advantages of tetraploid red clover cultivars compared to diploids are a higher concentration of proteins, water-soluble carbohydrates, potassium and phosphorus [42,43]. Moreover, tetraploid cultivars have more rapidly degradable proteins, lower lignin content and a lower level of unavailable carbohydrate fraction than diploids [44]. On the other hand, tetraploid red clover cultivars usually produce lower seed yields compared to diploids [43]. In this research, it can be observed that the yield of dry matter per ha of 2n red clover accessions in the second cut of the first year of exploitation ranged from 6.2 to 12.5 t/ha, while 4n red clover accessions achieved a higher yield than diploids, ranging from 9.6 to 13.9 t/ha (Table 1).

The influence of red clover ploidy on the isoflavone content and composition was investigated by only a few authors. Namely, Lemežienė et al. (2015) analyzed eight diploid and three tetraploid red clover cultivars, and no statistically significant impact of ploidy on isoflavone content was observed [37]. Similarly, Tsao et al. (2006) investigated seven diploid and six tetraploid cultivars and found that the average total isoflavone content of 2n samples was 17.15 mg/g and in 4n samples it was 17.27 mg/g; therefore, no significant difference was determined [7]. On the other hand, as mentioned in the Results section of this study, statistical analysis confirmed that tetraploid genotypes contain significantly higher levels of daidzein, genistein and formononetin compared to diploids. Namely, the total average isoflavone content in 4n samples is 3.67 ± 3.38 mg/g compared to 2.48 ± 2.15 mg/g of isoflavones in 2n. These findings, obtained on a larger sample than the previous studies, imply that ploidy could still be the factor which determines isoflavone content.

Characteristics of certain plant genotypes could be related to their geographical origin. Also, specific traits, such as isoflavone content, can be transmitted to the plant offspring [45]. Consequently, it can be assumed that these traits might be retained even if the plants are grown in different localities. However, in this study, a clear statistical distinction between red clover samples obtained from seeds of different origin was not established, although it can be noticed that the genotypes from Slovakia, Germany and Sweden have higher average isoflavone content compared to the samples from France and the Czech Republic. This could be the consequence of the same environmental and climate conditions in which all of the samples were cultivated. Namely, it was previously established that the isoflavone content can be influenced by periods of sunlight, current weather conditions, soil characteristics and many other abiotic factors [4]. For example, in a paper published by Sazdanić et al. (2018), statistical analysis showed that red clover samples grown in different countries are grouped based on isoflavone content. More specifically, samples originating from Lithuania and Serbia belonged to the same group, while samples originating from Brazil and Finland were both separated. It was pointed out that ecological characteristics of habitat, with an emphasis on temperature and precipitation, are influencing factors on the content of these secondary metabolites [46]. Also, it is likely that varieties with a lower isoflavone content were created through selection in order to prevent disruption of the reproductive cycle in sheep caused by a high concentration of isoflavones. Considering the previously mentioned facts, it could be summarized that isoflavone production in red clover depends on complex interactions and a combination of certain genetic and environmental factors.

Horvat et al. (2020) analyzed leaf samples at the full flowering stage of 29 diploid red clover varieties from Croatia. The values obtained for TPC were from 38.67 to 59.96 mg GAE/g of dry matter (with an average value of 49.12 ± 4.94 mg GAE/g) [47]. The results for the total phenolic content of leaf samples in this study range from 7.35 to 38.38 mg GAE/g (Table 3), which is lower compared to the mentioned Croatian samples. Also, leaves from diploid genotypes (on average 25.07 mg GAE/g) had generally lower TPC values than the 4n samples (on average 33.31 mg/GAE/g). However, the total phenolic content obtained for the Croatian red clover leaves [47] is comparable to values of TPC of flower extracts in this study. Furthermore, values of TPC of aerial parts of in vitro and in vivo grown red clover plants were 16.90–31.95 and 27.57–46.88 mg GAE/g, respectively [48]. Similarly, the TPC in red clover from Ireland sampled between April and August was in the whole plant between 38.6 and 47.49 mg GAE/g [39]. In this study, the content of total phenolics for the whole plant can be expressed as an average value of TPC obtained for leaf, flower and stem, and it is 21.8 ± 6.12 mg GAE/g, which is in the same range as the values obtained in the previous paper for the in vitro grown samples [39].

## 5. Conclusions

Red clover is a valuable source of phytoestrogens, with biochanin A and formononetin as major compounds accounting for more than 65% of total isoflavones. Observing different plant parts, it was determined that there are significant differences concerning all of the examined traits. Namely, leaves were most abundant in isoflavones (5.96 ± 3.04 mg/g), while flowers had the most potent antioxidant activity (IC_50_ 0.099 ± 0.035 mg/mL). When the obtained data were analyzed according to the sample ploidy, it was shown that the total isoflavone content in the leaves and flowers of 4n samples was over 50% higher compared to its content in 2n samples (in leaves 7.52 compared to 4.96 mg/g; in flowers 2.32 compared to 1.43 mg/g). Additionally, 2n and 4n genotypes were significantly different concerning their daidzein, genistein, formononetin and TPC content. The recorded variability of samples with different ploidy mostly correlated with biochanin A and genistein content and with antioxidant potential, while daidzein is the compound responsible for the separative grouping of 2n and 4n genotypes. According to the seeds’ country of origin, red clovers with seeds originating from Slovakia (3.61 ± 2.94 mg/g) and Germany (3.45 ± 2.18 mg/g) had the highest total isoflavone content, while the lowest isoflavone content was detected in the samples originating from France (1.42 ± 1.18 mg/g). However, there was no separative grouping of the studied samples according to this characteristic. It could be concluded that the isoflavone content in red clover depends on a complex interaction of genetics and environment with plant ploidy as a newly found and potentially important factor that should be further investigated. These results could contribute to the selection and breeding of cultivars with a high isoflavone content suitable for application in the food or pharmaceutical industry.

## Figures and Tables

**Table 1:** Red clover genotypes—ploidy and seed origin, with dry matter yield (t per ha) in the second cut of the first year of exploitation.

Samples	Sweden	Germany	Slovakia	Serbia	Czech Republic	France
Diploid (2n)	m20 (Bombi) 10.7	m33 (Atelo) 8.6	m77 (Marieta) 9.5	k 17 8.7	m84 (Bohemia) 7.3	m58 (Crop) 8.1
	m23 (Merkur) 8.6	m34 (Heges H) 8.5	m79 (Slatina) 12.5	Kolubara 8.1	m86 (Chlumecky II) 7.2	m59 (Diper) 7.2
			m80 (Viglana) 5.7	Una 8.6	m90 (Radan) 6.2	m62 (Levezou) 7.9
				Avala 8.5	m91 (Slovenska B) 8.3	m63 (Pales) 8.7
						m65 (Verdi) 7.6
Tetraploid (4n)	m21 (Fanny) 10.7	m36 (Jubilatka) 11.9	m81 (Sigord) 12.2			
	m24 (Molly) 13.9	m40 (Matero) 10.9	m82 (Margot) 11.6			
	m26 (Pelly) 9.6	m41 (Matri) 10.4				
	m28 (Sally) 11.4					
	m29 (Sara) 13.2					

**Table 2:** Isoflavone content (mg/g of DW) in different plant parts of red clover genotypes.

		Leaf					Flower					Stem				
Origin	Genotype	D *	G	F	B	Total	D	G	F	B	Total	D	G	F	B	Total
Sweden	m20	0.808	1.895	4.343	2.246	9.292	0.079	0.796	0.328	0.400	1.604	0.262	0.127	0.424	0.104	0.916
	m21	0.626	0.905	4.678	3.469	9.679	0.187	0.850	0.949	0.938	2.924	0.260	0.112	0.296	0.083	0.751
	m23	0.176	3.241	1.487	1.237	6.141	n.q.	0.703	0.193	0.331	1.227	0.160	0.704	0.798	0.231	1.894
	m24	0.531	1.524	2.920	3.436	8.411	0.100	0.864	0.558	0.550	2.072	0.242	0.180	0.371	0.115	0.909
	m26	0.365	1.142	1.281	0.876	3.664	0.129	0.469	0.335	0.342	1.274	0.289	0.219	0.362	0.174	1.043
	m28	0.239	1.303	1.739	1.804	5.084	0.076	0.973	0.651	0.887	2.587	0.141	0.157	0.277	0.125	0.700
	m29	0.198	1.063	1.380	1.005	3.646	0.065	0.518	0.279	0.174	1.036	0.310	0.221	0.293	0.084	0.909
Germany	m33	0.250	1.224	1.359	2.009	4.842	0.079	0.661	0.465	0.582	1.787	0.334	0.119	0.143	0.092	0.689
	m34	0.467	0.988	2.576	2.471	6.502	0.084	0.524	0.444	0.570	1.622	0.230	0.235	0.214	0.157	0.835
	m36	0.445	1.732	1.470	1.466	5.113	0.155	0.970	0.438	0.634	2.197	0.176	0.177	0.290	0.205	0.848
	m40	0.376	4.519	2.129	4.869	11.894	0.074	1.182	0.498	1.521	3.275	0.165	0.438	0.277	0.238	1.118
	m41	0.454	1.717	2.091	2.474	6.736	0.129	0.988	0.677	0.891	2.686	0.387	0.245	0.685	0.323	1.640
Slovakia	m77	0.243	0.627	0.305	0.591	1.766	0.045	0.507	0.139	0.315	1.005	0.116	0.511	0.237	0.184	1.048
	m79	0.328	0.597	1.061	1.742	3.727	0.121	0.504	0.584	1.081	2.289	0.347	0.242	0.600	0.229	1.417
	m80	0.264	1.781	2.892	3.528	8.465	0.132	1.228	0.620	1.004	2.983	0.501	0.411	0.515	0.257	1.683
	m81	0.460	2.777	1.328	3.378	7.943	0.096	0.798	0.532	1.153	2.580	0.590	0.613	0.531	0.398	2.131
	m82	0.428	2.623	4.506	5.495	13.051	0.080	0.770	0.780	0.904	2.534	0.519	0.307	0.466	0.242	1.533
Serbia	k17	0.287	0.818	2.169	1.629	4.903	0.138	0.371	0.260	0.422	1.191	0.318	0.117	0.440	0.147	1.021
	Kolubara	0.225	1.570	2.066	3.623	7.484	0.271	0.466	0.328	0.649	1.715	0.270	0.141	0.103	0.105	0.619
	Una	0.255	0.985	1.213	1.259	3.712	0.067	0.500	0.328	0.464	1.359	0.213	0.439	0.301	0.197	1.150
	Avala	0.128	1.397	1.964	1.863	5.352	0.095	0.408	0.331	0.473	1.307	0.174	0.521	0.443	0.273	1.411
Czech R.	m84	0.246	0.602	3.443	3.960	8.251	n.q.	0.321	0.636	0.759	1.717	0.373	0.213	1.544	0.565	2.695
	m86	0.189	0.611	1.064	2.293	4.157	n.q.	n.q.	n.q.	0.288	0.288	n.q.	n.q.	0.215	0.161	0.375
	m90	n.q.	0.157	0.544	0.984	1.684	n.q.	n.q.	n.q.	0.451	0.451	0.161	0.096	0.336	0.285	0.878
	m91	n.q.	0.224	0.374	0.794	1.393	n.q.	0.105	0.285	0.525	0.916	0.157	0.122	0.232	0.243	0.754
France	m58	n.q.	0.127	0.459	1.693	2.279	n.q.	0.111	0.212	0.875	1.198	n.q.	n.q.	0.115	0.174	0.288
	m59	n.q.	n.q.	0.277	0.593	0.870	n.q.	n.q.	0.274	0.504	0.778	n.q.	n.q.	0.193	0.186	0.378
	m62	n.q.	0.430	0.729	5.034	6.194	n.q.	n.q.	0.307	0.817	1.124	n.q.	0.128	0.228	0.254	0.611
	m63	n.q.	0.140	0.800	2.479	3.419	n.q.	0.192	0.116	0.593	0.901	0.171	0.117	0.421	0.207	0.915
	m65	n.q.	0.120	0.220	0.770	1.110	n.q.	0.090	0.110	0.520	0.720	n.q.	n.q.	0.100	0.450	0.550

In gray cells—4n genotypes; other—2n genotypes; * D—daidzein, G—genistein, F—formononetin, B—biochanin A; n.q.—not quantified.

**Table 3:** Antioxidant capacity (IC_50_) and total phenolic content (TPC) of different plant parts of red clover genotypes.

		IC_50_ (mg/mL)			TPC (mg GAE/g of DW)		
Origin	Genotype	Leaf	Flower	Stem	Leaf	Flower	Stem
Sweden	m20	0.152 ± 0.001	0.168 ± 0.007	0.377 ± 0.007	18.92 ± 0.25	17.51 ± 0.71	10.16 ± 0.54
	m21	0.143 ± 0.001	0.082 ± 0.006	0.228 ± 0.012	32.81 ± 0.21	39.50 ± 1.25	16.84 ± 0.22
	m23	0.106 ± 0.011	0.123 ± 0.004	0.379 ± 0.005	27.52 ± 0.46	23.96 ± 0.47	8.70 ± 0.74
	m24	0.132 ± 0.001	0.109 ± 0.021	0.465 ± 0.013	32.00 ± 0.50	30.65 ± 0.55	11.37 ± 0.14
	m26	0.140 ± 0.003	0.158 ± 0.005	0.430 ± 0.005	32.52 ± 0.19	22.69 ± 0.32	12.44 ± 0.58
	m28	0.114 ± 0.010	0.071 ± 0.006	0.568 ± 0.008	31.16 ± 0.36	43.18 ± 0.43	9.58 ± 0.41
	m29	0.176 ± 0.021	0.106 ± 0.008	0.436 ± 0.011	26.30 ± 0.82	29.26 ± 0.67	12.94 ± 0.89
Germany	m33	0.091 ± 0.003	0.074 ± 0.004	0.359 ± 0.060	31.00 ± 0.45	39.09 ± 0.52	10.88 ± 0.58
	m34	0.102 ± 0.006	0.085 ± 0.004	0.413 ± 0.101	29.16 ± 0.41	35.58 ± 0.71	11.08 ± 0.33
	m36	0.085 ± 0.003	0.098 ± 0.002	0.442 ± 0.017	35.91 ± 0.38	32.70 ± 0.25	10.46 ± 0.19
	m40	0.092 ± 0.008	0.059 ± 0.008	0.412 ± 0.019	38.38 ± 0.18	47.05 ± 0.68	9.54 ± 0.21
	m41	0.078 ± 0.002	0.066 ± 0.004	0.478 ± 0.008	37.23 ± 0.19	38.75 ± 0.20	9.61 ± 0.20
Slovakia	m77	0.073 ± 0.003	0.077 ± 0.001	0.400 ± 0.006	37.89 ± 0.80	31.21 ± 0.89	8.77 ± 0.14
	m79	0.077 ± 0.009	0.070 ± 0.002	0.360 ± 0.029	37.33 ± 0.22	38.19 ± 0.58	10.77 ± 0.22
	m80	0.109 ± 0.005	0.081 ± 0.001	0.354 ± 0.018	32.35 ± 0.33	35.45 ± 0.33	12.57 ± 0.36
	m81	0.077 ± 0.003	0.067 ± 0.003	0.319 ± 0.055	35.66 ± 0.29	37.18 ± 0.41	11.09 ± 0.41
	m82	0.096 ± 0.005	0.064 ± 0.007	0.290 ± 0.047	31.11 ± 0.24	40.72 ± 0.55	12.17 ± 0.14
Serbia	k17	0.078 ± 0.005	0.086 ± 0.004	0.398 ± 0.070	31.43 ± 0.43	28.68 ± 0.47	6.76 ± 0.22
	Kolubara	0.094 ± 0.003	0.074 ± 0.003	0.393 ± 0.011	28.05 ± 0.49	32.95 ± 0.59	8.18 ± 0.12
	Una	0.096 ± 0.004	0.082 ± 0.005	0.415 ± 0.046	31.98 ± 0.08	26.18 ± 0.74	6.24 ± 0.14
	Avala	0.088 ± 0.007	0.105 ± 0.003	0.333 ± 0.041	29.75 ± 0.13	25.36 ± 0.58	6.38 ± 0.15
Czech R.	m84	0.080 ± 0.006	0.093 ± 0.005	0.279 ± 0.012	26.20 ± 0.19	26.74 ± 0.45	9.50 ± 0.11
	m86	0.120 ± 0.004	0.145 ± 0.004	0.417 ± 0.018	27.66 ± 0.38	19.20 ± 0.36	10.25 ± 0.22
	m90	0.079 ± 0.004	0.167 ± 0.001	0.332 ± 0.022	27.77 ± 0.41	15.36 ± 0.25	7.75 ± 0.17
	m91	0.215 ± 0.005	0.196 ± 0.002	0.345 ± 0.006	18.67 ± 0.39	15.13 ± 0.14	9.32 ± 0.15
France	m58	0.208 ± 0.005	0.114 ± 0.008	0.396 ± 0.039	7.35 ± 0.13	13.68 ± 0.42	3.90 ± 0.11
	m59	0.120 ± 0.002	0.083 ± 0.006	0.312 ± 0.012	15.77 ± 0.49	17.61 ± 0.18	2.89 ± 0.12
	m62	0.092 ± 0.005	0.068 ± 0.001	0.290 ± 0.011	18.73 ± 0.33	22.35 ± 0.22	6.58 ± 0.14
	m63	0.182 ± 0.001	0.114 ± 0.003	0.337 ± 0.035	12.76 ± 0.39	13.39 ± 0.44	5.87 ± 0.12
	m65	0.112 ± 0.002	0.070 ± 0.001	0.307 ± 0.011	11.20 ± 0.14	16.22 ± 0.25	7.64 ± 0.23

In gray cells—4n genotypes; other—2n genotypes; average value ± st.dev. (*n* = 3).

## Data Availability

Data is contained within the article or supplementary material.

## Supplementary Materials

The following supporting information can be downloaded at https://www.mdpi.com/article/10.3390/foods13010103/s1, Figure S1: A—chromatogram of mixture of standard compounds (dzi-daidzein tr = 19.2 min, gen-genistein tr = 30.0 min, form-formononetin tr = 38.3 min, bioch-biochanin A tr = 42.5 min); B—chromatogram of sample m21 leaf extract.

## References

[1] Ergon A., Bakken A.K. (2022). Breeding for intercropping: The case of red clover persistence in grasslands. Euphytica.

[2] McKenna P., Cannon N., Conway J., Dooley J. (2018). The use of red clover (*Trifolium pratense*) in soil fertility-building: A review. Field Crop. Res..

[3] Petrauskas G., Norkevičienė E., Baistruk-Hlodan L. (2023). Genetic differentiation of red clover (*Trifolium pratense* L.) cultivars and their wild relatives. Agriculture.

[4] Little V., Reed K.F.M., Smith K.F. (2017). Variation for concentrations of various phytoestrogens and agronomic traits among a broad range of red clover (*Trifolium pratense*) cultivars and accessions. Agronomy.

[5] Mustonen E.A., Tuori M., Kurki P., Isolahti M., Taponen J., Vanhatalo A. (2018). Variety, time of harvest and conditions during growing season have impact on red clover isoflavone content. Agric. Food Sci..

[6] Sakakibara H., Viala D., Ollier A., Combeau A., Besle J.M. (2004). Isoflavones in several clover species and milk from goats fed clovers. Biofactors.

[7] Tsao R., Papadopoulos Y., Yang R., Young J.C., McRae K. (2006). Isoflavone content of red clovers and their distribution in different parts harvested at different growing stages. J. Agric. Food Chem..

[8] Saviranta N., Anttonen M., Wright A., Karjalainen R. (2008). Red clover (*Trifolium pretense* L.) isoflavones: Determination of concentrations by plant stage, flower colour, plant part and cultivar. J. Sci. Food Agric..

[9] Spanguolo P., Rasini E., Luini A., Legnaro M., Luzzani M., Casareto E., Carreri M., Paracchini S., Marino F., Cosentino M. (2014). Isoflavone content and estrogenic activity of different batches of red clover (*Trifolium pretense* L.) extracts: An in vitro study in MCF-7 cells. Fitoterapia.

[10] Andres S., Hansen U., Niemann B., Palavinskas R., Lampen A. (2015). Determination of the isoflavone composition and estrogenic activity of commercial dietary supplements based on soy or red clover. Food Funct..

[11] Budryn G., Gałązka-Czarnecka I., Brzozowska E., Grzelczyk J., Mostowski R., Żyżelewicz D., Cerón-Carrasco J.P., Pérez-Sánchez H. (2018). Evaluation of estrogenic activity of red clover (*Trifolium pratense* L.) sprouts cultivated under different conditions by content of isoflavones, calorimetric study and molecular modelling. Food Chem..

[12] Tian Z., Liu S.-B., Wang Y.-C., Li X.-Q., Zheng L.-H., Zhao M.-G. (2013). Neuroprotective effects of formononetin against NMDA-induced apoptosis in cortical neurons. Phytother. Res..

[13] Tay K.-C., Tan L.T.-H., Chan C.K., Hong S.L., Chan K.-G., Yap W.H., Pusparajah P., Lee L.-H., Goh B.-H. (2019). Formononetin: A review of its anticancer potentials and mechanisms. Front. Pharmacol..

[14] Feng Z.-J., Lai W.-F. (2023). Chemical and biological properties of biochanin A and its pharmaceutical applications. Pharmaceutics.

[15] Raheja S., Girdhar A., Lather V., Pandita D. (2018). Biochanin A: A phytoestrogen with therapeutic potential. Trends Food Sci. Technol..

[16] Petrović M.P., Stanković M.S., Anđelković B.S., Babić S.Ž., Zornić V.G., Vasiljević S.L., Dajić-Stevanović Z.P. (2016). Quality parameters and antioxidant activity of three clover species in relation to the livestock diet. Not. Bot. Horti. Agrobo..

[17] Tava A., Pecio Ł., Lo Scalzo R., Stochmal A., Pecetti L. (2019). Phenolic content and antioxidant activity in Trifolium germplasm from different environments. Molecules.

[18] Myers S.P., Vigar V. (2017). Effects of a standardised extract of *Trifolium pratense* (Promensil) at a dosage of 80 mg in the treatment of menopausal hot flushes: A systematic review and meta-analysis. Phytomedicine.

[19] Bhagwat S., Haytowitz D.B. (2015). USDA Database for the Isoflavone Content of Selected Foods, Release 2.1. http://www.ars.usda.gov/nutrientdata.

[20] Mikulić M., Atanacković Krstonošić M., Sazdanić D., Cvejić J., Li Y., Qi B. (2022). Health Perspectives on Soy Isoflavones. Phytochemicals in Soybean. Bioactivity and Health Benefits.

[21] Almeida I.M., Rodrigues F., Sarmento B., Alves R.C., Oliveira M.B. (2015). Isoflavones in food supplements: Chemical profile, label accordance and permeability study in Caco-2 cells. Food Funct..

[22] Maul R., Kulling S.E. (2010). Absorption of red clover isoflavones in human subjects: Results from a pilot study. Br. J. Nutr..

[23] Sivesind E., Seguin P. (2005). Effects of the environment, cultivar, maturity, and preservation method on red clover isoflavone concentration. J. Agric. Food Chem..

[24] Butkutė B., Lemežienė N., Dabkevičienė G., Jakštas V., Vilčinskas E., Janulis V. (2014). Source of variation of isoflavone concentrations in perennial clover species. Pharmacogn. Mag..

[25] Taujenis L., Padarauskas A., Mikaliūnienė J., Cesevičienė J., Lemežienė N., Butkutė B. (2015). Identification of isoflavones and their conjugates in red clover by liquid chromatography coupled with DAD and MS detectors. Chemija.

[26] Egan L.M., Hofmann R.W., Ghamkhar K., Hoyos-Villegas V. (2021). Prospects for trifolium improvement through germplasm characterisation and pre-breeding in New Zealand and Beyond. Front. Plant Sci..

[27] Řepková J., Nedělník J. (2014). Modern methods for genetic improvement of *Trifolium pratense*. Czech J. Genet. Plant Breed..

[28] Klejdus B., Vitamvasova D., Kuban V. (1999). Reversed-phase high-performance liquid chromatographic determination of isoflavones in plant materials after isolation by solid-phase extraction. J. Chromatogr. A.

[29] Krenn L., Unterrieder I., Ruprechter R. (2002). Quantification of isoflavones in red clover by high-performance liquid chromatography. J. Chromatogr. B.

[30] Kroyer G.T. (2004). Red clover extract as antioxidant active and functional food ingredient. Innov. Food Sci. Emerg. Technol..

[31] Brand-Wiliams W., Cuvelier M.E., Berset C. (1995). Use of a free radical method to evaluate antioxidant activity. Food Sci. Technol..

[32] Booth N.L., Overk C.R., Yao P., Totura S., Deng Y., Hedayat A.S., Bolton J.L., Pauli G.F., Farnsworth N.R. (2006). Seasonal variation of red clover (*Trifolium pratense* L., Fabaceae) isoflavones and estrogenic activity. J. Agric. Food Chem..

[33] Çölgeçen H., Çalişkan U.K., Kartal M., Büyükkartal H.N. (2014). Comprehensive evaluation of phytoestrogen accumulation in plants and in vitro cultures of *Medicago sativa* L. ‘Elçi’ and natural tetraploid *Trifolium pratense* L.. Turk. J. Biol..

[34] Ercetin T., Toker G., Kartal M., Colgecen H., Toker M.C. (2012). In vitro isoflavonoid production and analysis in natural tetraploid *Trifolium pratense* (red clover) calluses. Rev. Bras. Farmacogn..

[35] Gikas E., Alesta A., Economou G., Karamanos A., Tsarbopoulos A. (2008). Determination of isoflavones in the aerial part of red clover by HPLC–Diode Array Detection. J. Liq. Chromatogr. Relat. Technol..

[36] Hloucalová P., Skládanka J., Horký P., Klejdus B., Pelikán J., Knotová D. (2016). Determination of phytoestrogen content in fresh-cut legume forage. Animals.

[37] Lemežienė N., Padarauskas A., Butkutė B., Cesevičienė J., Taujenis L., Norkevičienė E., Mikaliūnienė J. (2015). The concentration of isoflavones in red clover (*Trifolium pratense* L.) at flowering stage. Zemdirb. Agric..

[38] Ramos G.P., Dias P.M., Morais C.B., Fröehlich P.E., Dall’Agnol M., Zuanazzi J.A. (2008). LC determination of four isoflavone aglycones in red clover (*Trifolium pratense* L.). Chromatographia.

[39] Rapisarda S., Abu-Ghannam N. (2023). Polyphenol characterization and antioxidant capacity of multi-species swards grown in Ireland—Environmental sustainability and nutraceutical potential. Sustainability.

[40] Radinović I., Vasiljević S., Zorić M., Branković G., Živanović T., Prodanović S. (2018). Variability of red clover genotypes on the basis of morphological markers. Genetika.

[41] Sattler M.C., Carvalho C.R., Clarindo W.R. (2016). The polyploidy and its key role in plant breeding. Planta.

[42] Drobna J., Jančovič J. (2006). Estimation of red clover (*Trifolium pratense* L.) forage quality parameters depending on the variety, cut and growing year. Plant Soil Environ..

[43] Amdahl H., Aamlid T.S., Marum P., Ergon Å., Alsheikh M., Rognli O.A. (2017). Seed yield components in single plants of diverse Scandinavian tetraploid red clover populations (*Trifolium pratense* L.). Crop Sci..

[44] Marković J., Lazarević Đ., Bekčić F., Terzić D., Vasić T., Živković S., Štrbanović R. (2022). Protein and carbohydrate profiles of a diploid and a tetraploid red clover cultivar. Agric. Food Sci..

[45] Miladinović J., Đorđević V., Belešević-Tubić S., Petrović K., Ćeran M., Cvejić J., Bursać M., Miladinović D. (2019). Increase of isoflavones in the aglycone form in soybeans by targeted crossings of cultivated breeding material. Sci. Rep..

[46] Sazdanić D., Mikulić M., Kladar N., Hogervorst J., Atanacković Krstonošić M. (2018). Analysis of the factors influencing red clover (*Trifolium pratense* L., Fabaceae) isoflavone content. Biol. Serb..

[47] Horvat D., Tucak M., Viljevac Vuletić M., Čupić T., Krizmanić G., Kovačević Babić M. (2020). Phenolic content and antioxidant activity of the Croatian red clover germplasm collection. Poljoprivreda.

[48] Esmaeili A.K., Taha R.M., Mohajer S., Banisalam B. (2015). Antioxidant activity and total phenolic and flavonoid content of various solvent extracts from in vivo and in vitro grown *Trifolium pratense* L. (Red clover). BioMed Res. Int..

