# Supplementary material for: Phytochemical Composition of Different Red Clover Genotypes Based on Plant Part and Genetic Traits
Source: Foods. 2023 Dec 28;13(1):103. doi: 10.3390/foods13010103 (PMC10778848; doi:10.3390/foods13010103)
Supplement: Supplementary file 1 [file foods-13-00103-s001.zip › foods-2764618-supplementary.pdf]

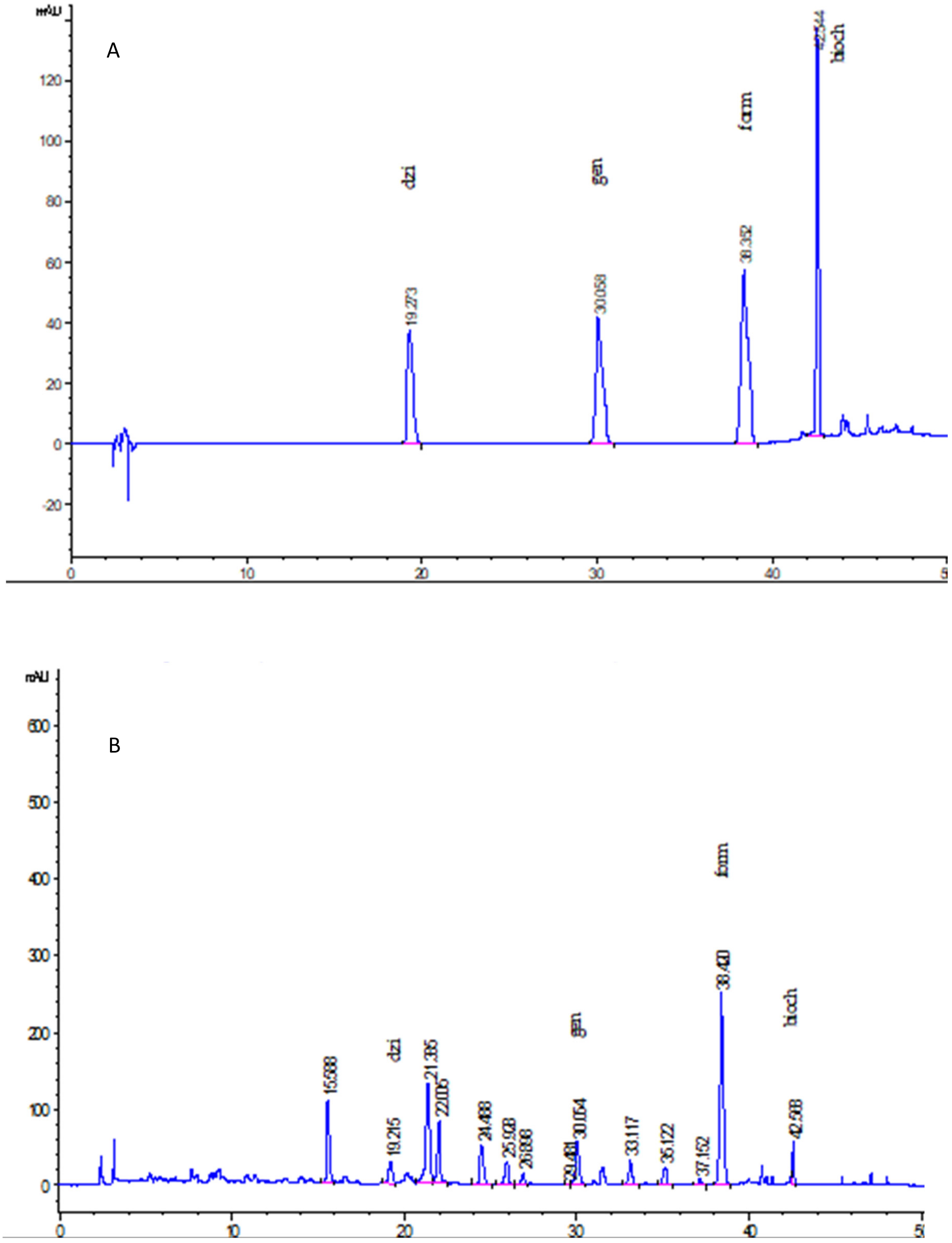

Figure S1.

A – chromatogram of mixture of standard compounds (dzi-daidzein t_r=19.2 min, gen-genistein t_r=30.0 min, form-formononetin t_r=38.3 min, bioch-biochanin A t_r=42.5 min);

B – chromatogram of sample m21 leaf extract
